# Supplementary material for: Frictional healing and induced earthquakes on conventionally stable faults
Source: Nat Commun. 2025 Oct 15;16:9140. doi: 10.1038/s41467-025-63482-3 (PMC12528417; doi:10.1038/s41467-025-63482-3)
Supplement: Supplementary file 1 — Supplementary Information [file 41467_2025_63482_MOESM1_ESM.pdf]

Supplementary information

Frictional healing and induced earthquakes on conventionally stable faults

Meng Li*, Andre R. Niemeijer, Ylona van Dinther

Department of Earth Sciences, Utrecht University, Princetonlaan 4, 3584 CS, Utrecht, Netherlands

*Corresponding author: limeng.uni@gmail.com

This document includes

Supplementary Figures 1-5

Supplementary Table 1

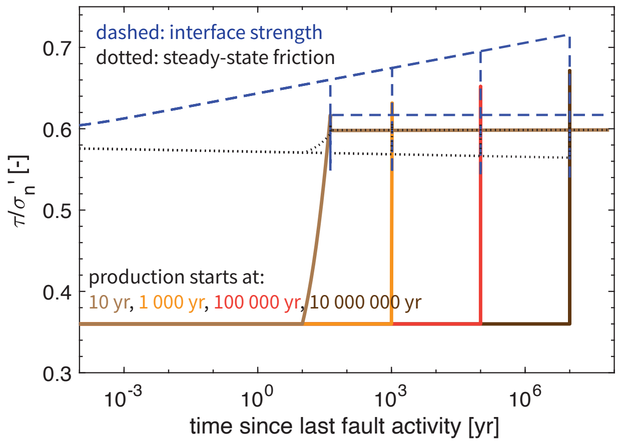

Supplementary Fig. 1 Evolution of fault friction and interface strength over geological time scales. Simulation of earthquake sequences in VS scenarios ($a = 0.013$) with different lengths of healing time from 10 yr to 100 Ma. The ratio of shear stress and effective normal stress τ/σ_n' is plotted with respect to the time of the event since the last fault activity, i.e., where healing starts. The interface strength (Eq. 1, in blue) and steady-state friction (Eq. 18, in black) are for reference.

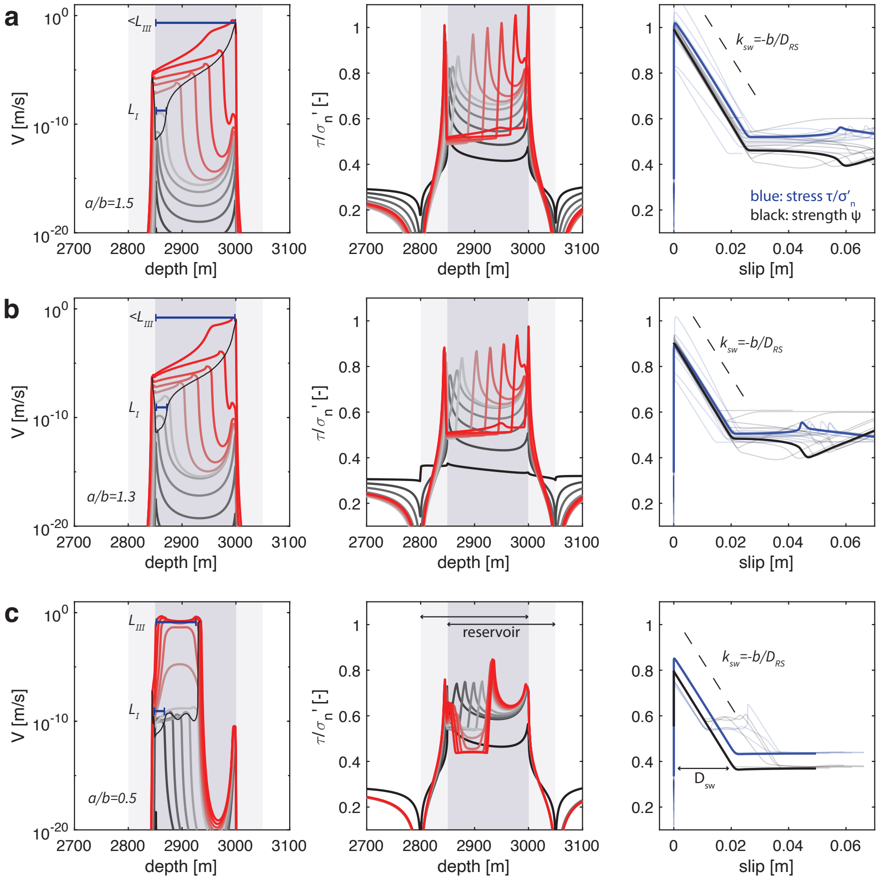

Supplementary Fig. 2 Simulation of the first induced earthquake in 2-D in VS and VW scenarios with several a/b ratios: (**a**) $a/b = 1.5$, (**b**) $a/b = 1.3$, (**c**) $a/b = 0.5$ with fixed $b = 0.01$ and $D_{RS} = 0.5$ mm, $\mu_0 = 0.5$, healing time $t_h = 100$ Ma. The temporal evolution of slip rate, the ratio between the shear stress and the effective normal stress, and its evolution with respect to slip are plotted. The interseismic and nucleation phases are plotted in black and gray, with gradual transit to the coseismic phase in red. The plotted lines are not picked up in regular time intervals. The measured nucleation lengths L_I, L_{II} and L_{III} are shown as blue bars on the top. The dashed black lines track the temporal-spatial evolution of the nucleation front. The purple shadows in the background specify the range of the reservoir depth, on the hanging wall side and the footwall side, respectively. The detailed model setup is depicted in Fig. 4. The evolution of interface strength Ψ (Eq. 1) is plotted in black for reference. The multiple transparent lines in the background are the observations from different locations (every 20 m between 2800 m and 3050 m depth) on the fault. The bold lines are the observation at the center of the reservoir (2950 m depth).

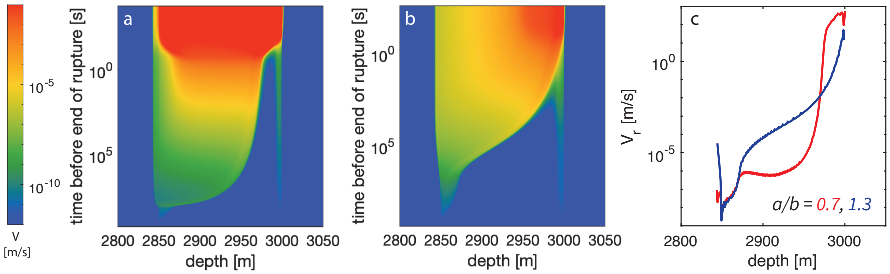

Supplementary Fig. 3 Nucleation of the first induced earthquake in 2-D in VS and VW scenarios with (**a**) $a/b = 0.7$, (**b**) $a/b = 1.3$, with fixed $b = 0.01$ and $D_{RS} = 0.5$ mm, $\mu_0 = 0.5$, healing time $t_h = 100$ Ma. The spatial-temporal evolution of slip rate is shown in color. (**c**) Measured propagation speed of the nucleation front for $a/b = 0.7$ (red) and $a/b = 1.3$ (blue).

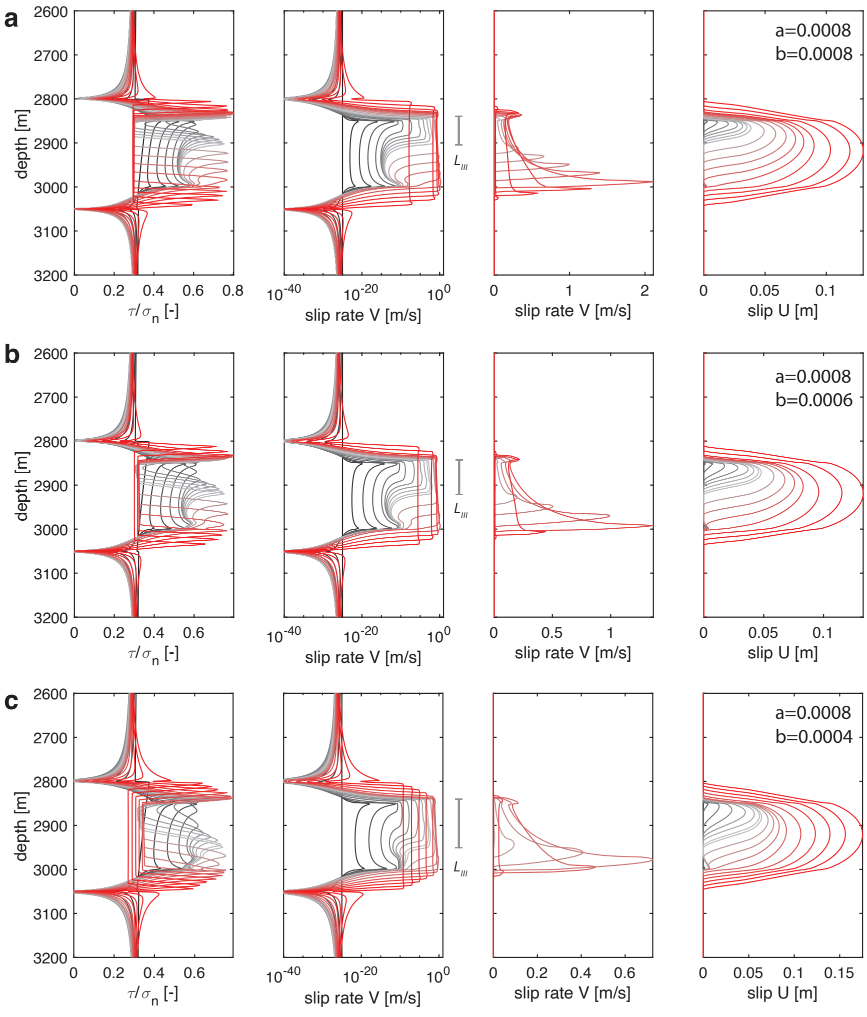

Supplementary Fig. 4 Simulation of the first induced earthquake in 2-D with different b values. $a = 0.0008, \mu_0 = 0.3$ and other parameters are kept fixed. The interseismic and nucleation phases are plotted in black and gray, with gradual transit to the coseismic phase in red. The plotted lines are not picked up in regular time intervals. The nucleation zones are highlighted by the gray bars.

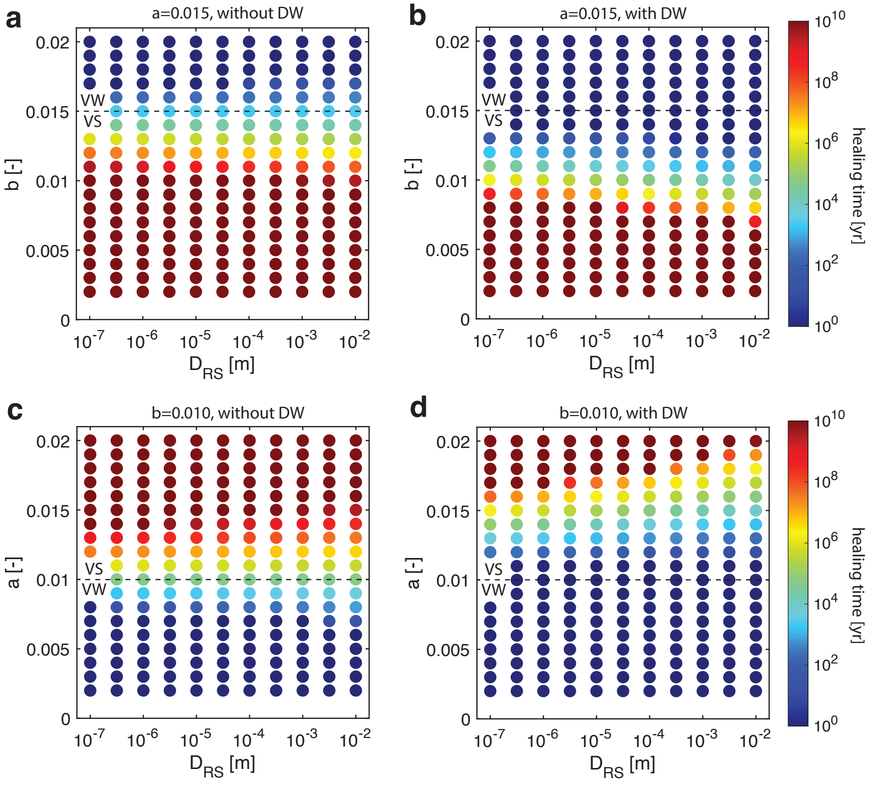

Supplementary Fig. 5 Parameter study of frictional properties and healing time: (**a-b**) the parameter study on b and D_{RS}, (**c-d**) the parameter study on a and D_{RS}, (a, c) without dynamic weakening, (b, d) with dynamic weakening. The colored circles show the required healing time so that the first earthquake, after fault reactivation, can reach a slip rate of 1 m/s.

Supplementary Table 1 Physical and numerical parameters

Parameter	Symbol	Value
Density		
- rock (sandstone)	ρ_r	2400 kg/m^3
- fluid	ρ_f	1150 kg/m^3
- gas	ρ_g	200 kg/m^3
Shear wave speed in rock	c_s	1.645 km/s
Poisson ratio of rock	ν	0.15
Biot coefficient	α	1
Reference friction coefficient	μ_0	0.6 (default), 0.3-0.6
Reference slip rate	V_0	10^{-6} m/s
Characteristic slip distance[a]	D_{RS}	0.5 mm (default), 0.1 mm-0.01 m
Rate-and-state direct effect[a]	a	0.002-0.02
Rate-and-state evolution effect[a]	b	-0.002-0.02
Dynamic weakening velocity (Eq. 11)	V_w	1 m/s
Far-field loading rate	V_p	0 m/s
Pressure depletion rate[b]	$\dot{P}$	-0.0211 Pa/s
Loading path factor (0-D model)[c]		
- on normal stress	γ_n	-0.2818
- on shear stress	γ_s	-0.4469

[a] The parameter range used this study, specific values are mentioned in figure captions.
[b] Based on the average production rate of Groningen gas field since 1961 [48].
[c] Simulated values extracted from the 2-D model.
